# Reversed better-than-average effect in direct comparisons of nonsocial stimuli depends on the set size

**DOI:** 10.3758/s13421-013-0385-7

**Published:** 2013-12-19

**Authors:** Jakub Niewiarowski, Jerzy J. Karyłowski, Karolina Szutkiewicz-Szekalska, Marzena Cypryańska

**Affiliations:** 1 Department of Psychology, University of Social Sciences and Humanities, Chodakowska 19/31, 03-815 Warsaw, Poland; 2 Institute of Psychology, Polish Academy of Sciences, Warsaw, Poland; 3 University of North Florida, Jacksonville, FL USA; 4 University of Social Sciences and Humanities, Warsaw, Poland

**Keywords:** Better-than-average effect, Set size, Absolute judgment, Comparative judgment, Nonsocial stimuli, Reversed effect

## Abstract

Studies on direct comparative judgments typically show that, for items that are positively evaluated, a single item randomly drawn from a larger set of similar items tends to be judged as better than average (the BTA effect). However, Windschitl, Conybeare, and Krizan (2008) demonstrated that, under timing conditions that do not favor focusing attention on the single item, the reversal of the BTA effect occurs. We report two experiments showing that the magnitude of the reversed BTA effect increases as a function of the size of a multiitem referent with which a single item target is compared. Specifically, in direct comparative judgments of the attractiveness of positively evaluated objects (nice-looking cloth buttons, attractive buildings, or cupcakes), underestimation of the attractiveness of singletons, as compared with a multiitem set (reversed BTA effect), increased with the increased set size. Analysis of absolute judgments obtained for singletons and for small and large multiitem sets suggests that, for attractive stimuli, both the reversed BTA effect in comparative judgments and its sensitivity to set size occur as a result of a positive relationship between set size and perceived attractiveness in absolute judgments.

Having to make a comparison between a specific item and a set of remaining items constitutes a common dilemma. Is this box of chocolates higher quality than most, so it deserves a higher price? Is this used car in a better condition than a typical used car in this price range and should therefore be further considered? Is this job applicant clearly better than an average candidate in the pool and should be selected for an interview? Each of these questions calls for a direct comparative judgment between a specific item (singleton) and a multiitem set from which that specific item was drawn.

There is extensive empirical evidence that such comparisons can lead to biased judgments in which the extremity of a singleton is overemphasized. For items that are positively evaluated, a single item is typically judged as better than average (the BTA effect). Thus, in the social domain, a randomly selected individual is typically judged as having more positive characteristics than the group average (e.g., Klar, 2002; Klar & Giladi, 1997, 1999; Suls, Lemos, & Lockett, 2002). This occurs even when knowledge concerning that individual is very limited—for instance, when the individual is denoted by an identification number only (Klar & Giladi, 1997). Furthermore, in making comparisons between self (singleton) and others (multiple-item set), people typically see themselves as more likely than average to experience events that are relatively common and less likely than average to experience events that are relatively rare (Chambers, Windschitl, & Suls, 2003; Kruger & Burrus, 2004). They also believe that they perform better than average on tasks that are relatively easy and less than average on difficult tasks (Kruger, 1999). Thus, in making direct comparisons between individuals and groups, individuals are typically judged as more extreme than the groups, more likely to experience common events and possess common characteristics, and less likely to experience uncommon events and possess uncommon characteristics.

Experimental evidence for overemphasizing the extremity of a singleton involved in direct comparisons with multiitem sets is not limited to social objects. Specifically, for positively evaluated nonsocial objects (such as liked songs, healthy food, or pleasant-smelling soaps), single items tend to be judged as more positive than the average of the remaining items from the same group. For instance, when participants were asked to smell six pleasant-smelling soaps and were subsequently presented with a randomly chosen soap from that set and were asked to judge its smell, as compared with the average of the five remaining soaps, they typically judged the singleton as having a more pleasant smell than the average of the remaining soaps. The opposite pattern occurred for sets of unpleasant-smelling soaps (Giladi & Klar, 2002, Experiment 1).

A notable exception to this pattern of results comes from research by Windschitl, Conybeare, and Krizan (2008). Using photographs of nonsocial objects such as sofas, hotel rooms, or vacation spots that were preselected as highly attractive, they obtained a reversal of the usual BTA effect. Specifically, in making direct comparisons between a single item and a four-item set, participants judged the singletons as less attractive than the rest of the set.^1^ This occurred even when singletons were denoted as targets and multitarget sets were denoted as referents, a condition normally conducive to obtaining classic BTA effects (cf. Chambers & Windschitl, 2004; Suls, Chambers, Krizan, Mortensen, Koestner, & Bruchmann, 2010).

In explaining the reversal of the usual BTA effect, Windschitl and colleagues (2008) speculated that the experimental procedures used in previous studies tended to increase the relative salience, at the time the judgment was made, of the single-item object rather than the multiitem set involved in the comparison. For instance, participants in Giladi and Klar’s (2002) experiments were first familiarized with a set of items (e.g., soaps) and only subsequently were presented with a randomly selected singleton (e.g., a specific soap) and asked to make a comparative judgment. Thus, it is likely that at the time the judgment was made, the singleton was more salient for the participants than the remaining items involved in the comparison. Because salient objects tend to be judged as more extreme (see Hagemann, Strauss, & Leising, 2008; Kardes, Sanbonmatsu, Cronley, & Houghton, 2002), the BTA/worse-than-average (WTA) effects for positive and negative items, respectively, would be expected.

In a related finding, Windschitl et al. (2008) showed that in indirect comparisons—that is, when participants made separate attractiveness judgments of attractive sets and singletons—attractiveness judgments were lower for singletons than for the sets. This finding is, of course, consistent with the results they obtained for direct comparisons. If, judged separately, singletons are seen as less attractive than multiitem sets, other things being equal (e.g., absence of strong enough factors enhancing relative salience of singletons, as compared with such sets), singletons should be judged as less attractive also in direct comparisons with multiple-item sets, which is exactly what Windschitl et al. found. While the exact reasons for results involving indirect comparisons are not entirely clear (cf. Windschitl et al., 2008), the reversal of the usual BTA effect for singletons when they are directly compared with multiitem sets, in the absence of critical factors increasing the salience of singletons, would be expected.

In the present experiments, we had two principal goals. First, we attempted to provide a conceptual replication of the reversed BTA effect for direct comparisons involving attractive singletons and multiitem sets. Second, we examined the impact of the size of the multiitem set on the magnitude of the effect.

Previous attempts to address the impact of set size have been limited to experimental procedures conducive to producing classic BTA/WTA effects, and not the reversed effect reported by Windschitl et al. (2008). Moreover, the results of such attempts have been inconclusive. Thus, Price, Smith, and Lench (2006, Experiment 2) reported that the tendency to see the self as less vulnerable to relatively uncommon negative life events increased as a logarithmic function of the size of the comparison group. Yet Suls et al. (2010) found no reliable impact of the size of the comparison group on the classic BTA/WTA effect for either positive or negative and either social or nonsocial stimuli.

With respect to the reversed BTA effect, we propose that, for attractive items, a larger size of the multiitem set used in direct comparison would be associated with a greater tendency to judge the singleton as worse than the set. Thus, a stronger reversed BTA effect should be observed. We believe that this prediction follows from at least two arguments. First, a larger set should be more likely to include items that constitute particularly strong examples of whatever is the predominant focal characteristic of the entire sample—for instance, items that are particularly attractive. Because such stimuli tend to attract attention, this “pick the best” strategy (Szutkiewicz-Szekalska & Niewarowski, in press; Windschitl et al., 2008) would result in more pronounced disadvantage of singletons over larger sets. Second, as described by the sample size bias hypothesis (Smith & Price, 2010), a larger set size may be confused by observers with a more extreme standing on a relevant dimension. For instance, in estimating a mean value of a set of numbers, people typically give more inflated estimates for larger than for smaller sets (Smith & Price, 2010). Similarly, stimuli that are larger in size tend to be judged as more attractive than smaller stimuli (Silvera, Josephs, & Giesler, 2002). Finally, in estimating the average height of a set of human figures, people give higher estimates in the case of larger than in the case of smaller sets (Price et al., 2006, Experiment 5). This happens even when all figures included in a given set are of the same height, thus excluding the “pick the best” strategy as the sole underlying mechanism. In any case, the question of the underlying mechanism notwithstanding, the reversed BTA effect, as demonstrated by Windschitl et al., should be more pronounced for larger than for smaller sets involved in direct comparisons with singletons.

We tested this prediction in two experiments modeled on the direct comparison procedure used in Windschitl et al. (2008, Experiment 2). However, unlike in the original experiment, the size of the multiitem referents that participants used in making direct comparisons with singleton targets was, in the present experiments, systematically varied.

## Experiment 1

### Method

#### Participants

One hundred fifty-four Polish college students (106 women, 48 men) participated in the experiment.

#### Stimuli

We took photographs of 60 cloth buttons bought at a local haberdashery store. Photographs were digitally processed to remove differences in the size of the buttons. Attractiveness of each button was assessed on a 5-point Likert-type scale by an independent sample of 77 students. Fifty attractive buttons with means higher than the scale midpoint were selected as stimuli for the experiment.

#### Design and procedure

Participants were tested individually in a computer lab. There were 38 trials, each calling for a direct comparative judgment of attractiveness of a single-item target, as compared with a referent consisting of a set of 2, 4, 8, 16, or 24 items. Of the 38 trials, there were 10 each involving 2-item, 4-item, and 8-item referents. In addition, there were 6 trials involving referents consisting of 16 items and 2 trials involving referents consisting of 24 items. The order of trials using referents of a different set size was counterbalanced across participants.

**Fig. 1:**
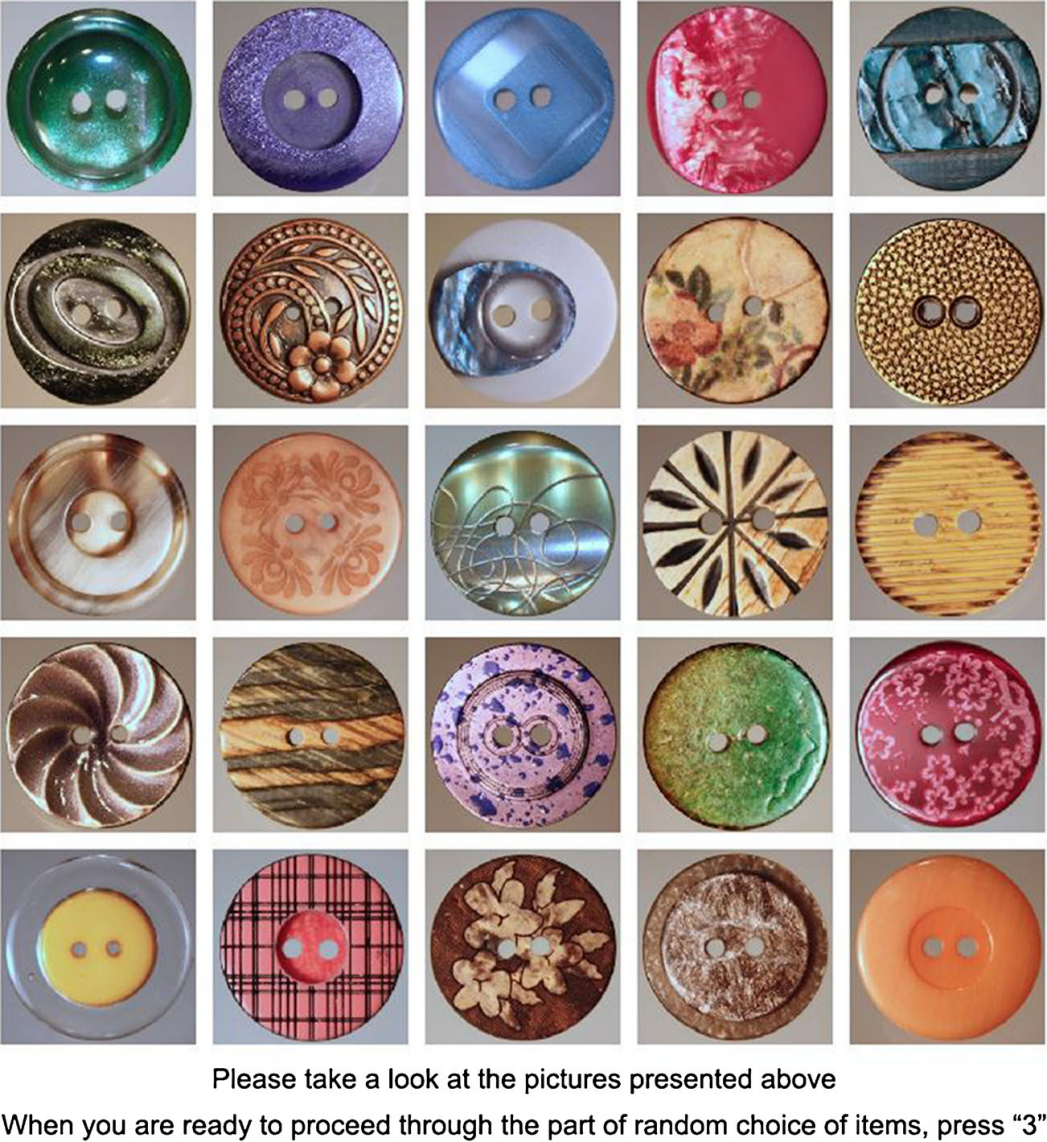
An example of how the initial 25-item matrix appeared on the computer screen before participants proceeded through the part of random choice of the target and referent group

**Fig. 2:**
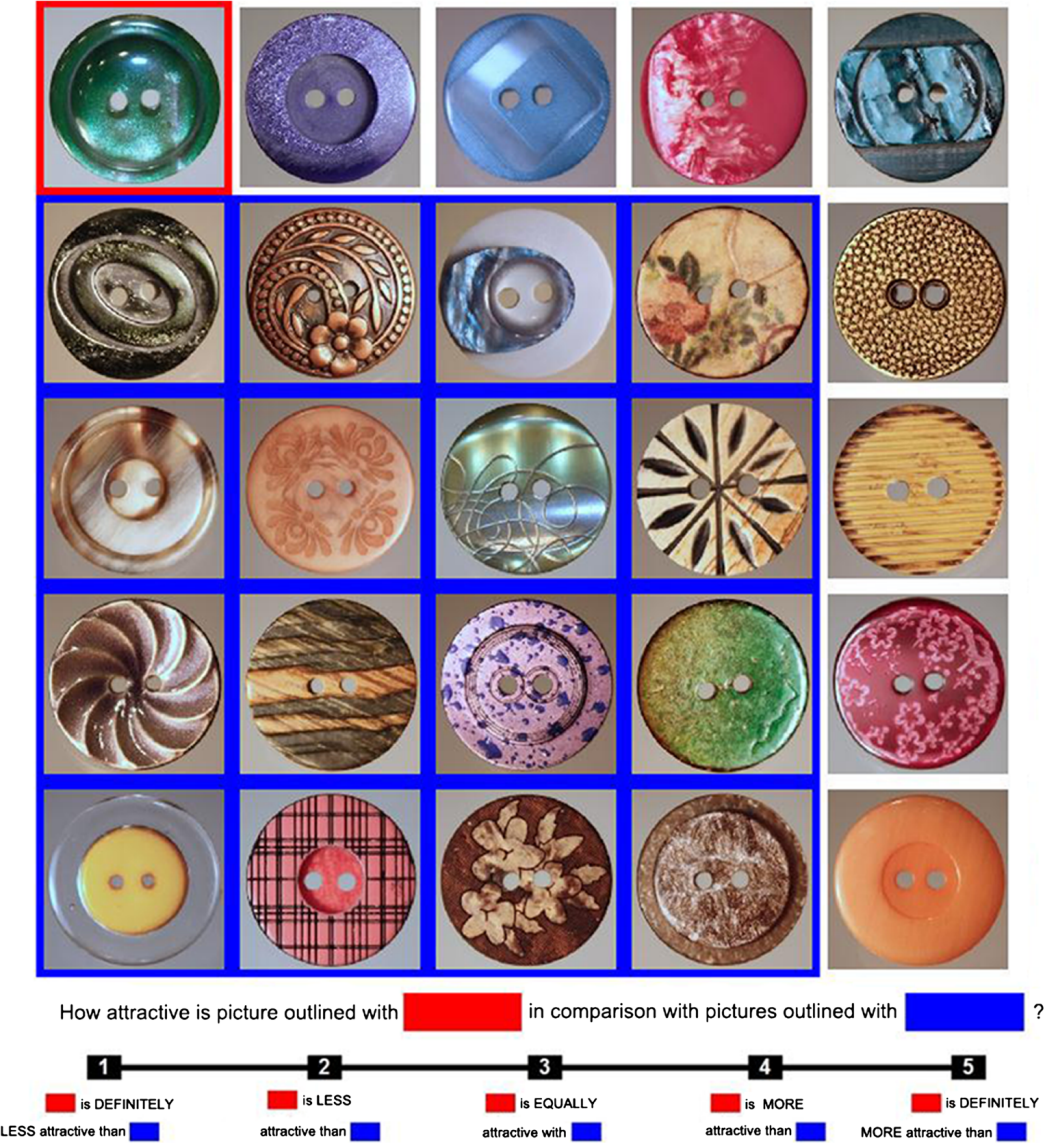
An example of how the participants were asked the comparative question. This figure presents 1 versus 16 comparisons in the context-present condition. The target is denoted with red, and the referent group with blue

At the onset of each trial, participants were presented on a computer screen with a 5 × 5 matrix consisting of 25 pictures (items), each representing a different cloth button. Two sets of pictures, each consisting of 25 items, were used throughout the experiment, alternating between odd- and even-numbered trials and counterbalanced across participants. On each consecutive odd or even trial, the position of pictures forming the matrix was rotated, with the rotation order counterbalanced across participants. The initial presentation of the 5 × 5 matrix was always accompanied by the following instruction appearing at the bottom of the screen: “Please take a look at the pictures presented above. When you are ready to proceed to the random choice of items, press 3” (see Fig. 1). At the pressing of the “3” key, the initial instruction was replaced with the following comparative judgment question: “How attractive is the picture outlined in ‘red’ in comparison with pictures outlined in ‘blue’?” (where “red” and “blue” were appropriately colored rectangles). A 5-point scale ranging from 1 = “*red*” *is definitely less attractive than* “*blue*” to 5 = “*red*” *is definitely more attractive than* “*blue*” was presented below. After an additional 500 ms, the target and referent were indicated by means of outlining the target with red and the referent with blue (see Fig. 2). On each trial, the singleton target and the multiitem referent were randomly selected and outlined with appropriate colors. The target was always selected out of the two items occupying the upper left corner and the lower left corner of a given matrix. The referent was always selected from the remaining items, with the stipulation that items forming the referent were adjacent (and thus, formed a figure that could be outlined). Participants entered their judgments by pressing numerical keys “1” through “5.”

There were five types of comparisons depending on the referent size (i.e., 1 vs. 2, 4, 8, 16, or 24). The number of repeats of each type of comparison depended on the size of the referent: Comparisons of 1 versus 2, 4, or 8 were repeated 10 times each; comparisons of 1 versus 16 were repeated 6 times; and 1 versus 24 was repeated twice. Each participant gave answers on 38 trials. The order of the type of comparison was fully counterbalanced across participants.

For exploratory purposes, half of the participants were randomly assigned to the context-present condition, and the rest to the context-absent condition. In all comparison types, except 1 versus 24, only part of the matrix was covered by outlined pictures. In the context-absent condition after the target and referent were designated, items not outlined were removed from the screen at the time of answering, so the only items participants saw belonged to the particular comparison. In the context-present condition, all the items (outlined and not outlined) were present during comparison.

### Results and discussion

For each participant, we computed mean comparative assessment scores across trials with specific referent set sizes (i.e., 2, 4, 8, 16, 24) and a specific target position (upper vs. lower), arriving at 10 mean comparative assessments.

**Fig. 3:**
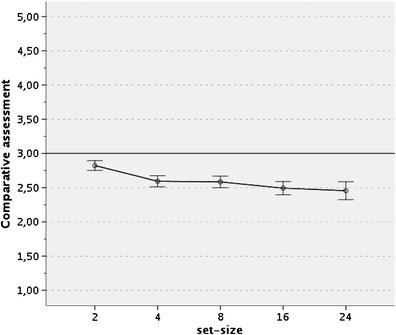
Mean comparative attractiveness judgments as a function of the referent set size. Judgments were made on a 5-point comparative scale, where 1 indicated *preference for the multiitem set* (referent) and 5 indicated *preference for the singleton* (target). Scores below scale midpoint (3) indicate preference for the multiitem set

As is shown in Fig. 3, for each of the set sizes, assessments were lower than the midpoint of the direct comparative scale, indicating that in direct comparative judgments, single-item targets were evaluated less positively than the multiitem referents. A series of single-sample *t*-tests revealed that the effect was significant for each of the set sizes, all *p*s < .001. This replicates the reversed BTA effect found for four-item sets by Windschitl et al. (2008) and extends the effect to the other set sizes used in the present experiment.

To test our main prediction that the magnitude of the reversed BTA effect would increase as a function of the referent set size, we first performed a 5 (referent set size: 2 vs. 4 vs. 8 vs.16 vs. 24)  ×  2 (target position: upper vs. lower)  ×  2 (context: absent vs. present) multivariate mixed-model ANOVA with the last factor as a between-subjects variable. As was expected, the main effect for the referent set size was significant, *F*(4, 149)  =  11.70, *p* <  .001, partial η^2^  =  .24, indicating that the magnitude of the reversed BTA effect depended on the set size of the referent. No other significant effects emerged. The main effect of referent group size was then tested using the trend analysis, revealing a significant linear trend, *F*(1, 152)  =  29.96, *p* <  .001, partial η^2^  =  .16. Figure 3 presents the means of comparative assessments for each of the referent sizes. As can be seen, the reversed BTA effect occurred for each of the referent set sizes: All means fell below a midpoint of the direct comparative scale, thus indicating preference for the multiitem referent over the single-item target. Furthermore, as was predicted, the larger the set size, the stronger the preference was.^2^

## Experiment 2

### Overview

Experiment 2 had two goals. First, we wanted to replicate the results of Experiment 1, using different types of stimuli and different modes of presentation. Second, we attempted to extend the results of Experiment 1 to indirect comparisons based on separate attractiveness judgments of singletons and multiitem sets. This would provide indirect evidence that both the reversed BTA effect in direct comparisons and the sensitivity of this effect to the referent set size are due to the increased attractiveness of larger sets.

### Method

#### Participants

One hundred sixty Polish college students (104 women, 56 men) participated in the experiment.

#### Stimuli

Fifteen photographs of buildings (architectural monuments) and 15 photographs of cupcakes were used as stimuli. The stimuli were selected from larger sets of relatively unfamiliar photographs on the basis of attractiveness ratings provided by two independent samples of 72 students (buildings) and 90 students (cupcakes). A 5-point scale was used to collect judgments of buildings, and a 7-point scale was used to collect judgments of cupcakes. For each set, only photographs with mean ratings above the scale’s midpoint were used in the experiment.

#### Design and procedure

Participants were tested individually in a computer lab. The experiment was divided into two stages. During the first stage, participants made direct comparative judgments of attractiveness involving single-item targets and three-item or eight-item referents. During the second stage, the attractiveness of each of the single-item and multiitem stimuli used during the first stage was judged separately.

1. The first stage consisted of 20 trials divided into four blocks of 5 trials each. Two different referent set sizes (three vs. eight) were used in the odd-numbered and the even-numbered blocks and were counterbalanced across participants. Also, two different types of stimuli (buildings vs. cupcakes) were used during the first two and the second two blocks, again counterbalanced across participants. At the onset of each trial, participants were presented on a computer screen with a 5 × 3 (columns and rows, respectively) matrix consisting of 15 pictures of cupcakes or 15 pictures of buildings, each representing a different stimulus. Within each of the four blocks, the position of the pictures forming the matrix for each of the 5 trials was rotated, with the rotation order counterbalanced across participants. All other aspects of the procedure during the first stage were the same as in Experiment 1, except for the following:For half of the participants, the target and referent always occupied an adjacent position and together formed a 2 × 2 or a 3 × 3 matrix (for three-item and eight-item referents, respectively). For the remaining participants, the target and referent were separated by at least one other item. This manipulation was included for exploratory purposes.
2. In addition, for half of the participants, the target was always outlined in red and the referent was always outlined in green, while for the other half, this was reversed. This was done to eliminate a potential source of confounding present in Experiment 1.
3. The target was always randomly selected out of the entire 15-item matrix, thus making its location less predictable.
4. No initial instruction asking participants to look at the pictures and to press a key when ready to proceed was presented.
5. No context manipulation was included in the design; for each trial, all 15 items remained on the screen for the duration of the trial.

The second stage of the experiment consisted of 20 pairs of trials, each calling for an absolute judgment of either target or referent used in one of the 20 comparative judgments collected during the first stage and presented in the same order as during the first stage. For each of the original target/referent combinations, two absolute judgments were made in consecutive trials, with the order (target first vs. referent first) counterbalanced across participants. On each trial, the original stimulus (target or referent) was presented as part of the same 5 × 3 matrix as that during the first stage; however, only the target or only the referent was outlined on a given trial. The colors used to outline the targets and referents were the same as during the first stage (green for targets and red for referents or red for targets and green for referents). Judgments were made on a scale from 1 (*very unattractive*) to 5 (*very attractive*) in response to the following question: “How attractive is (are) the building (buildings) [or the cupcake (cupcakes)] marked with red [green]?”

### Results and discussion

#### Direct comparative judgments

For the first stage of the experiment, for each participant, we computed mean direct comparative assessment scores across trials with specific referent set-sizes (i.e., three vs. eight) and specific stimulus type (buildings vs. cupcakes), thus arriving at four scores.

As is shown in the upper panel of Table 1, for larger set sizes, for both buildings and cupcakes, assessments were significantly lower than the midpoint of the direct comparative scale, indicating that single-item targets were evaluated less positively than the multiitem referents, thus replicating the reversed BTA effect found by Windschitl et al. (2008) and in our Experiment 1. The same tendency could be observed for smaller set sizes, but the effects were not significant.

**Table 1:** Mean judgments regarding stimuli by the within-subjects conditions of the Experiment 2

	Referent Size		
	3	8	Significance (Set Size Effect)
Direct Comparison Judgments			
Buildings	2.94	2.78**	*p* < .01
Cupcakes	2.97	2.88*	*p* = .075
Overall	2.95	2.83**	*p* < .005
Absolute Judgments: Targets			
Buildings	3.31**	3.27**	n.s.
Cupcakes	3.37**	3.37**	n.s.
Overall	3.35**	3.33**	n.s.
Absolute Judgments: Referents			
Buildings	3.44**	3.82**	*p* < .001
Cupcakes	3.50**	3.89**	*p* < .001
Overall	3.47**	3.87**	*p* < .001

*Note*. Direct comparative judgments were scored from 1 to 5. Values below midpoint (3) indicate preference for the multiitem set. Asterisks indicate significant deviation from the midpoint (target being equally attractive as referent), by a one-sample *t*-test. Absolute judgments were scored from 1 to 5, with higher numbers indicating greater perceived attractiveness of a stimulus. Asterisks indicate significant deviation from the midpoint (moderately attractive) by a one-sample *t*-test. Last column reflects the significance of the difference between means

*p < .05

**p < .001

To test the prediction that the magnitude of the reversed BTA effect would be greater for larger than for smaller sets, we performed a 2 (referent set size: three vs. eight) × 2 (material: buildings vs. cupcakes) × 2 (target/referent placement: adjacent vs. separated) multivariate mixed-model ANOVA with the last factor as a between-subjects variable. Consistent with our predictions and with the results of Experiment 1, the preference for multiitem referents over singleton targets was more pronounced—further below the midpoint—for larger (*M* = 2.83) than for smaller (*M* = 2.95) referents, resulting in the significant main effect of the referent set size, *F*(1, 158) = 9.73, *p* < .005, partial η^2^ = .06. This effect occurred regardless of the material (buildings vs. cupcakes) and regardless of the target/referent placement (adjacent vs. separated); neither interaction approached significance. The only additional effect approaching significance was an unexpected main effect of the material, *F*(1, 158) = 2.95, *p* = .09, partial η^2^ = .02, indicating that the preference for referents over targets was somewhat more pronounced for buildings (*M* = 2.86) than for cupcakes (*M* = 2.92).

#### Absolute judgments

For each participant, we used absolute judgments collected during the second stage to compute mean absolute judgment scores for singletons and multiitem sets from the first stage. This was done for three-item and eight-item trials and for buildings and cupcakes, separately. A 2 (stimulus type: singletons vs. multiitem sets) × 2 (referent set size: trials with three-item referents vs. trials with eight-item referents ) × 2 (material: buildings vs. cupcakes) repeated measures ANOVA performed on mean absolute judgments revealed a main effect of stimulus type, *F*(1, 158) = 116.84, *p* < .001, partial η^2^ = .42, a main effect of referent set size, *F*(1, 158) = 75.54, *p* < .001, partial η^2^ = .32, and a stimulus type × referent set size interaction, *F*(1, 158) = 75.92, *p* < .001, partial η^2^ = .32. As can be seen in Table 1, consistent with the reversed BTA effect found for direct comparative judgments, absolute judgments were lower for singleton targets than for multiitem referents. This was true for both three-item and eight-item referents, but the effect for the eight-item referents was stronger, *F*(1, 158) = 12.2, *p* < .01, partial η^2^ = .07, and *F*(1, 158) = 169,53, *p* < .001, partial η^2^ = .52, respectively. As would be expected, the main effect of the referent set size was due entirely to higher absolute judgments for multiple-item sets consisting of eight items than for such sets consisting of three items, *F*(1, 158) = 122,95, *p* < .001, partial *η* ^2^ = .44. Absolute judgments of singletons were not affected by whether they came from trials with three-item or eight-item referents, *F*(1, 158) < 1, n.s.

This pattern of results is fully consistent with the notion that, for attractive stimuli, both (1) the reversed BTA effect in direct comparative judgments and (2) its sensitivity to the size of a multiitem referent are due to increased attractiveness of larger sets, with singletons judged as least attractive. Because, on the basis of absolute judgments, singletons are perceived as less attractive than multiitem sets and because multiitem sets composed of fewer items are perceived as less attractive than sets composed of larger number of items, with no other intervening factors, the reversed BTA effect would be expected. Furthermore, such an effect should be stronger for larger than for smaller sets, the pattern observed in both our experiments.

## General discussion

In making direct comparative judgments, participants in the present experiments judged singletons as less attractive than multiitem sets. This occurred for different modes of presentation and for different types of stimuli. Because the stimuli were preselected as relatively attractive (above the scale midpoint), it may be concluded that the singletons were evaluated as less extreme than the multiitem sets. This result constitutes a conceptual replication of the reversed BTA effect demonstrated by Windschitl et al. (2008). The reversed effect occurred even though (similar to Windschitl et al., 2008, Experiment 2) participants made judgments with singletons denoted as targets and multiitem sets denoted as referents, a condition often considered as facilitating the occurrence of the usual (nonreversed) BTA effect (e.g., Chambers & Windschitl, 2004; Suls et al., 2010).

Admittedly, both the present demonstration of the reversed effect and the previous demonstration by Windschitl et al. (2008) are limited to judgments of attractiveness of stimuli preselected as relatively attractive. Whether a parallel reversed effect can be found for unattractive stimuli remains to be demonstrated. Similarly, future research should attempt to examine reversed effects in comparative judgments involving attributes other than attractiveness (e.g., frequency, size, probability, etc.).

Another potential limitation of present research that is shared with the previous demonstration of the reversed BTA effect by Windschitl et al. (2008) concerns the wording of instructions for making comparisons between singletons and multiitem sets. Specifically, no explicit reference was included to using the average of the set in making comparative judgments. Instead, participants were simply asked to make a comparison between the singleton and the set. Perhaps including such specific reference to relying on averages could discourage participants from using the “pick-the-best” strategy, thus reducing or eliminating the effect.

Notwithstanding those limitations, our results clearly show that the reversed BTA effect, as originally demonstrated by Windschitl et al. (2008), is sensitive to the size of the multiitem set used as a referent: The magnitude of the effect increases with the set size. In Experiment 1, when the design permitted testing for linearity of the relationship between the magnitude of the reversed BTA effect and the set size, the relationship was shown to be linear.

Data from Experiment 2 regarding absolute (noncomparative) judgments for singletons and multiitem sets that were used as targets and referents in direct comparative judgments in that experiment shed light on why the reversed BTA effect occurs (when it does) and why it is stronger for larger set sizes. The data show that (for attractive stimuli) judged attractiveness increases with the set size. Thus, singletons are judged as less attractive than multiitem sets, and smaller multiitem sets are judged as less attractive than larger multiitem sets.

Our finding that the magnitude of the reversed BTA effect in direct comparative judgments increases with the increased set size brings to mind results obtained by Price et al. (2006, Experiment 2). Those authors showed that direct comparative judgments regarding vulnerability of self, as compared with others, decreased with the number of others involved in the comparison. In relating the present results to the results obtained by Price et al., it might be helpful to distinguish between two aspects: (1) overestimation versus underestimation of extremity in judgments of singletons, as compared with multiitem sets, and (2) the impact of the set size on the magnitude of such over- or underestimation.

With respect to the first aspect, Price et al. (2006, Experiment 2) obtained the classic effect: more extreme judgments for singletons. Specifically, relatively unlikely events (becoming an alcoholic, breaking a bone, or getting cancer) were judged in direct comparisons as less likely (thus, more extreme) for the self (singleton) than for the average group member (multiitem set). In contrast, the reversed effect was demonstrated in the present experiments, thus replicating the results of Windschitl et al. (2008, Experiments 1, 2, and 3). Specifically, our results show that relatively attractive singletons were judged as less attractive (less extreme) when compared with the attractiveness of a multiitem set. In this respect, our results are opposite to the results obtained by Price et al.

With respect to the second aspect, our results are parallel to those in Price et al. (2006, Experiment 2) and show that the reversed effect is subject to a similar set size bias as the classic effect: In both cases, larger set sizes are associated with larger effects. Yet those findings are in contrast to the null results reported by Suls et al. (2010) for both social and nonsocial stimuli. While any interpretation of null results is, of course, problematic, it should be noted that Suls et al. (2010) used stimuli that were participant generated and highly familiar. In contrast, stimuli used by Price et al. and stimuli used in the present experiments were experimenter generated and relatively unfamiliar. This might suggest that high familiarity of stimulus materials limits bias due to the set size, perhaps because processing of highly familiar material requires fewer cognitive resources. Another, less interesting possibility is that the null results reported by Suls et al. (2010) reflect relatively low power resulting from manipulating the set size variable between participants. In contrast, both Price et al. and the present authors employed within-participants manipulations (with sample sizes similar to those in Suls et al., 2010).

Clearly, not enough is known about conditions leading to classic versus reversed BTA effects. While the present experiments were not designed to tackle this problem, our results are consistent with the notion that the classic effect may occur as a result of situational factors that facilitate focusing attention on the singleton just before the judgment is made. In particular, as was suggested by Windschitl et al. (2008), presenting a singleton target only after the multiitem referent is presented may force focusing attention on the singleton. This could make the singleton more vivid at the time the comparative judgment is made, leading to the classic BTA effect. Similar to Windschitl et al. (2008, Experiments 1, 2, 3), such timing conditions focusing attention on the singletons were not present in either of our experiments, perhaps contributing to a successful replication of the reversed BTA effect.

Obviously, factors other than timing might have been responsible for the increased attention to singletons, as compared with multiitem sets, contributing to the occurrence of the classic BTA effect in many previous studies. In particular, comparing self (singleton) with others (multiitem set) seems likely to give the singleton an attentional advantage because of the high chronic accessibility of the self (Chambers & Windschitl, 2004; Markus, 1980; Rogers, 1981). Indeed, numerous studies have found classic BTA/WTA effects for self–others comparisons (e.g., Aclicke, Klotz, Breitenbecher, Yurak, & Vredenburg, 1995; Brown, 2012; Chambers et al., 2003; Kruger, 1999; Kruger & Burrus, 2004; More & Small, 2007; Price et al., 2006).^3^

In any case, the results obtained in Experiment 2 for absolute (noncomparative) judgments showing that singletons, when judged separately, were seen as less attractive than multiitem sets suggest that it is the reversed, and not the classic, BTA effect that should be expected in direct comparative judgments when no additional situational or chronic factors leading to increased attention to the singletons are present. Furthermore, the pattern showing lower absolute judgments for smaller than for larger multiitem sets is fully consistent with our finding that the magnitude of the reversed BTA effect in direct comparative judgment increases with the increased size of the multiitem set. Thus, we believe that findings regarding the relationship between set size and absolute judgments advance our understating of the nature of the reversed BTA effect and its dependency on the size of the multiitem set. Yet the nature of the relationship between set size and absolute judgments is not entirely clear. Future research will undoubtedly focus on the contribution of various mechanisms responsible for effects regarding absolute judgments, including the “pick-the-best” strategy (Szutkiewicz-Szekalska & Niewiarowski, in press; Windschitl et al., 2008) and the sample size bias (Smith & Price, 2010).

## Footnotes

1 Such reversal of the classic BTA (better-than-average) effect should be distinguished from the classic WTA (worse-than-average) effect. The classic WTA effect occurs when, compared to multi-item sets, singletons are judged as lower on a given dimension (e.g., attractiveness) *for stimuli that*, *in general*, *are relatively low on that dimension* (e.g., are relatively unattractive). Thus, in the case of the classic WTA effect singletons are judged as relatively more extreme (same as is the case in the classic BTA effect). In contrast, the reversed BTA effect occurs when, compared to multi-item sets, singletons are judged as lower on a given dimension, *for stimuli that tend to be high on that dimension* (e.g., are relatively attractive). Thus, in this case, singletons are judged as relatively less extreme, compared to multi-item sets. Similarly, higher judgments for singletons compared to multi-item sets obtained for stimuli that are relatively low on a given dimension (e.g., relatively unattractive) would indicate the reversal of the classic WTA effect.

2 This effect of a referent set-size in direct-comparative judgments might be assessed differently. In their research, Price and colleagues (2006) state that different types of judgments tend to increase as a roughly logarithmic function of the size of a group. We have followed their strategy to analyze data within a regression approach (see full procedure in Lorch & Myers,^1990^). First we computed mean comparative assessment across trials with different referent set sizes (ignoring other factors, nonsignificant in omnibus ANOVA), then for each participant we regressed their five mean comparative assessments onto the natural logarithm of the referent set-size, thus obtaining single regression coefficients for each participant. As expected, the average regression coefficient (M = −.66; SD = 1.6) was significantly lower than 0, indicating an overall effect of a referent set-size, t(153) = 5.01, p < .001. The comparative assessments decreased as a function of a logarithm of a referent set- size.

3 Interestingly, simply denoting singleton as target does not seem to give it enough advantage over multi-item referent to produce the classic effect: both in Windschitl et al., (2008, Experiment 2) and in the present experiments, reversed BTA effect was observed in spite of designating singletons as targets and multi-item sets as referents.
